# Registration of Partially Focused Images for 2D and 3D Reconstruction of Oversized Samples

**DOI:** 10.1155/2017/8538215

**Published:** 2017-08-10

**Authors:** Dalibor Martišek, Hana Druckmüllerová

**Affiliations:** Faculty of Mechanical Engineering, Institute of Mathematics, Brno University of Technology, Technická 2, 616 69 Brno, Czech Republic

## Abstract

Methods of fracture surface 3D reconstruction from a series of partially focused images acquired in a small field of view (e.g., by confocal microscope or CCD camera) are well known. In this case, projection rays can be considered parallel and recorded images do not differ in any geometrical transformation from each other. In the case of larger samples (oversized for microscope or CCD camera), it is necessary to use a wider viewing field (e.g., standard cameras); taken images primarily differ in scaling but may also differ in shifting and rotation. These images cannot be used for reconstruction directly; they must be registered; that is, we must determine all transformations in which the images differ and eliminate their effects. There are several ways to do this. This paper deals with the registration based on phase correlation.

## 1. Introduction

**Figure 1:**
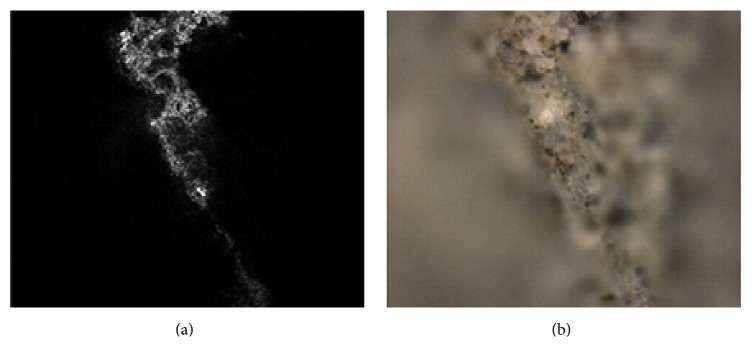
Fracture surface of cement paste. The image was acquired by confocal microscope in confocal mode (a) and nonconfocal mode (b).

This paper follows up recent published works [1, 2] about 3D reconstruction methods for images acquired by confocal and nonconfocal microscopes. Confocal microscopes are actually optical microscopes which are distinguished from other optical microscopes by two unique properties. They have a very small depth of optical field and their advanced hardware is capable of removing nonsharp points from images. The points of objects very near to the focal plane are visible as sharp points and are depicted as light areas in our optical sections (see Figure 1(a)) whereas those parts lying above or beneath the focal plane are invisible and are represented by black regions. Analogous regions can be observed by using conventional microscopes fitted with the same lens (see Figure 1(b)). Both the confocal and nonconfocal snapshots (optical sections) show sharp regions of very similar shapes. The only difference concerns nonsharp regions that manifest themselves as blurred regions in the nonconfocal sections, whereas in the confocal sections they are missing (black regions). However, the shapes of confocal and nonconfocal out-of-focus regions are very similar.

To create a sharp image (2D reconstruction), it is necessary to obtain a series of images of the same object, each of them with different focusing and each point on the object focused in one of the images (in the ideal case). The sharp parts are identified and composed in a new image. There is also a simple method for constructing a rough 3D model of the object where all sharp points belonging to the same image have the same height.

**Figure 2:**
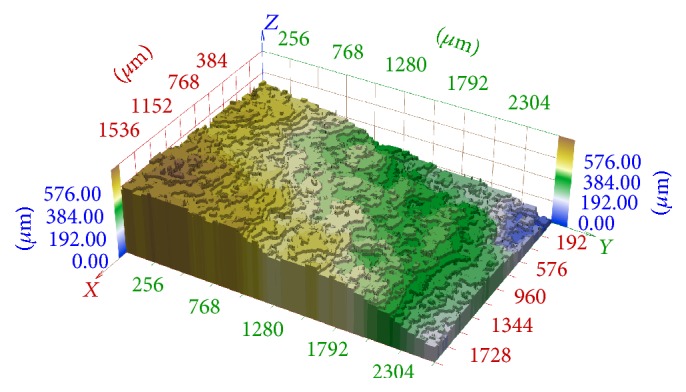
3D stair-approximation of fracture surface of hydrated cement paste.

**Figure 3:**
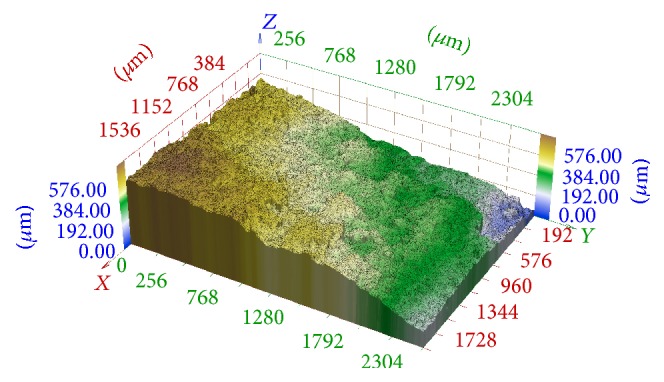
Smooth-approximation of fracture surface from Figure 2.

There are two principal problems in this 2D and 3D reconstruction. The work [3] deals with methods that detect focused and blurred parts in images taken in nonconfocal mode and are able to assign the corresponding focus plane for each surface point. In this way, construction of 3D stair-approximation of the studied surface is possible (see Figure 2). However, this approximation is not sufficient in many applications. It is necessary to specify the height of each point between two focal planes and construct a smooth approximation (see Figure 3).

**Figure 4:**
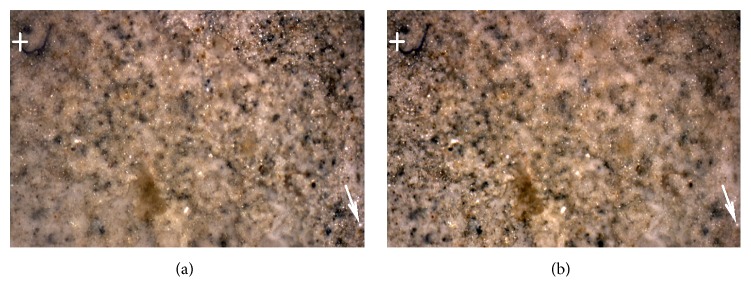
The first image (a) and the thirtieth image (b) in the series of photos of the fracture surface of hydrated cement paste acquired by confocal microscope Olympus LEXT 3100. The projection used is parallel and the fields of view are the same size (compare the position of the marked points).

**Figure 5:**
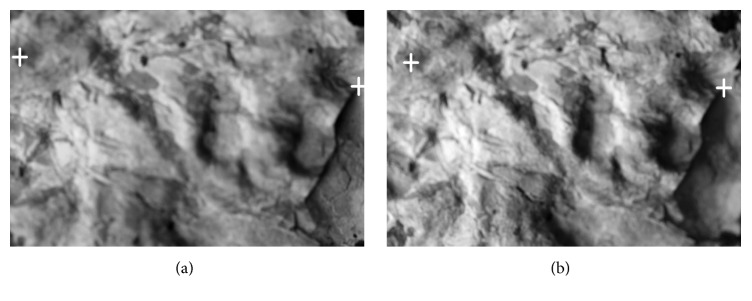
The first image (a) and the forty-third image (b) in the series of photos of sandstone sample (locality Brno-Hády, Czech Rep.) taken with a Canon DSLR camera. The projection used is central and the fields of view are clearly different (compare the position of the marked points).

The projection used is the second problem. In the case of the confocal microscope, we can assume that the field of view is small and the used projection is parallel. The paper [4] and many other works [3, 5–11] presume this projection property. In parallel projection, all images are provided in the field of view with the same size. In Figure 4, there is the first image (a) and the thirtieth image (b) in a series of fifty-two photos of the fracture surface of hydrated cement paste acquired by the confocal microscope Olympus LEXT 3100. Corresponding pixels have the same coordinates in separate partial focused images (compare the crosses and arrows in Figure 4). However, this assumption is not valid in the case of larger samples; the angle of the projection lines is not negligible and the view fields are different. In Figure 5, we can see the first image (a) and the forty-third image (b) in a series of seventy photos of a sandstone sample (locality Brno-Hády, Czech Republic) taken with a Canon DSLR camera. The used projection is central and the fields of view (i.e., also coordinates of corresponding pixels) of individual images are clearly different (see crosses in Figure 5).

Therefore, we set two goals in the conclusion of the work [1]: firstly, to specify the height of each point between two sharpness planes and construct a smooth approximation. This goal has already been met in [2]; for the result of this new reconstruction see Figure 3. Some other works [3, 8, 10] also deal with this problem.

The second goal, solution of different sizes of the field of view, is discussed in this paper.

## 2. Materials and Methods

### 2.1. Materials and Equipment

The methods discussed in this article are suitable for 2D and 3D processing of samples which are oversized for confocal microscopes. These samples may have a size from a few centimetres (e.g., geological samples). Data may be taken by a CCD camera or conventional digital camera with a narrow sharpness zone. This scanning device must be connected with a stepping device that allows changing the distance between the camera and the scanning sample and thus the position of the camera sharpness zone.

Data used in this paper was acquired by special hardware designed and assembled by professor Tomáš Ficker from the Faculty of Civil Engineering of our University. The hardware consists of a Canon EOS 600D photographic camera augmented by EF 100 mm f/2.8 Macro USM lens. The photographic camera is mounted on a motorized tough stand which enables movement in the vertical direction. The vertical stepping movement is governed by software running on a PC. See [12] for more details.

**Figure 6:**
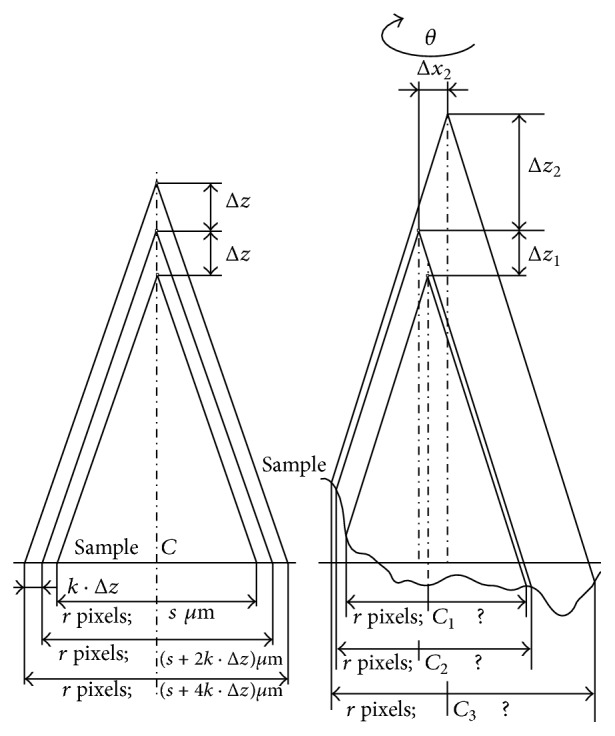
The central projection of an oversized sample, ideal case on the left, real case on the right.

Separate images are taken by central projection; that is, they differ at least in the scale used. The various scale of images could be solved using only elementary mathematics; the image size is proportional to the camera shifting in this case (see Figure 6 on the left).

However, the practical situation is more complicated. The images differ not only in the scale used but also in the content displayed (different parts are focused in different images). Due to mechanical errors, the step in the *z*-axis may be not fully constant; the images can also be mutually shifted in the *x*- or *y*-axis and even rotated. Image registration is also complicated by the nonplanarity of samples (see Figure 6 on the right). In Figure 5, we can see the first image (on the left) and the forty-third image (on the right) in a series of photos of a sandstone sample (locality Brno-Hády, Czech Republic) taken with a Canon DSLR camera. The projection used is central, the fields of view are clearly different.

In this paper we describe the preprocessing of a series of such images for 2D and 3D reconstruction. A suitable tool for this preprocessing is the Fourier transform.

### 2.2. Fourier Transform

This is an integral transform that transforms a function of one or more variables (in spatial domain) to another function (in frequency domain) of the same number of variables [3, 13, 14]. Since the Fourier transform of a function is in general a function with a complex image and since a digital image is a function of two spatial variables, we will deal here for simplicity with functions *f* : *ℝ*^2^ → *ℂ*.

Digital images are rectangles; for simplicity we deal here with square images only. All computations that use the Fourier transform are performed using the discrete Fourier transform (or more precisely by special algorithms that speed up the discrete Fourier transform, such as the Fast Fourier Transform (FFT)). However, some derivations of image processing methods are better performed with the Fourier transform of functions with the domain *ℝ*^2^ since operations such as rotation and rescaling are easily modeled on these functions.

The standard definition of the Fourier transform of a function of two variables is as follows [15].

**Definition 1 (Fourier transform).**

Let *f*(*x*; *y*) : *ℝ*^2^ → *ℂ* be a function such that

(1) $$\begin{array}{c} \overset{}{\underset{R^{2}}{\iint }}\left|\;f\left(\;x;y\;\right)\;\right|dx\;dy \end{array}$$

exists and is finite. The* Fourier transform* of *f* is

(2) $$\begin{array}{c} F\left(\;\xi ;\eta \;\right)=\overset{}{\underset{R^{2}}{\iint }}f\left(\;x;y\;\right)\exp\left(\;-i\left(\;x\xi +y\eta \;\right)\;\right)dx\;dy. \end{array}$$

Function *F*(*ξ*; *η*) is also called the Fourier spectrum of function *f*. Function *A*(*ξ*; *η*) = |*F*(*ξ*; *η*)| is called amplitude spectrum of *f*(*x*; *y*).

**Definition 2 (inverse Fourier transform).**

Let *F*(*ξ*; *η*) : *ℝ*^2^ → *ℂ* be a function such that

(3) $$\begin{array}{c} \overset{}{\underset{R^{2}}{\iint }}\left|\;F\left(\;\xi ;\eta \;\right)\;\right|d\xi \;d\eta \end{array}$$

exists and is finite. The* inverse Fourier transform* of function *F* is function

(4) $$\begin{array}{c} F^{-1}\left\{\;F\left(\;x;y\;\right)\;\right\}\left(\;x;y\;\right)=f\left(\;x;y\;\right):R^{2}⟶C \end{array}$$

defined as

(5) $$\begin{array}{c} f\left(\;x;y\;\right)=\frac{1}{4\pi^{2}}\overset{}{\underset{R^{2}}{\iint }}F\left(\;\xi ;\eta \;\right)\exp\left(\;i\left(\;x\xi +y\eta \;\right)\;\right)d\xi \;d\eta . \end{array}$$

### 2.3. Phase Correlation

For processing and analyzing the images it is necessary to transform the images so that the studied structures are at the same position in all the images. This is the task of image registration, to find the transformation. In some applications we assume that images were shifted only; in others we allow shift, rotation and scale change (i.e., similarity), general linear transformation, or even general transformations.

The methods used for registration depend on the expected transformation and on the structures in the image. Some methods use corresponding structures or points in the images and then find a global transformation using the measurements of positions of the structures or points [16–18]. These methods require these structures to be clearly visible. Other methods are based on correlation and work with the image as a whole. The phase correlation proved to be a powerful tool (not only) for registration of partially focused images. For functions *f*_1_; *f*_2_ it is defined as

(6) $$\begin{array}{c} P_{f_{1};f_{2}}\left(\;x;y\;\right)=F^{-1}\left\{\;\frac{F_{1}\left(\xi ;\eta \right)\cdot {\overset{-}{F}}_{2}\left(\xi ;\eta \right)}{\left|F_{1}\left(\xi ;\eta \right)\cdot F_{2}\left(\xi ;\eta \right)\right|}\;\right\} \end{array}$$

and its modification as

(7) Pf1;f2;;p;qx;y=F−1Hξ;η·F1ξ;η·F−2ξ;ηF1ξ;η+p·F2ξ;η+q,

where bar means complex conjugation and *H*(*ξ*; *η*) is a bounded real function such that *H*(*ξ*; *η*) = *H*(−*ξ*; −*η*) and *p*; *q* > 0 are arbitrary constants. It can be proved that for real functions *f*_1_; *f*_2_ the phase correlation function is real [14]. This is of great value, since it enables us to search for extremes of the phase correlation function.

### 2.4. Shifted Images

The phase correlation function can be also used for estimation of image shift. The method was first published by Kuglin and Hines [19].

It is clearly seen that the phase correlation function of a function with itself is the *δ*-distribution, that is,

(8) Pf;fx;yF−1Fξ;η·F−ξ;ηFξ;η·Fξ;η=F−11=δx;y

*δ*-distribution is a generalized function for which

(9) δx;y=∞;x;y=0;00;x;y≠0;0,∬R2δx;ydx dy=1.

**Figure 7:**
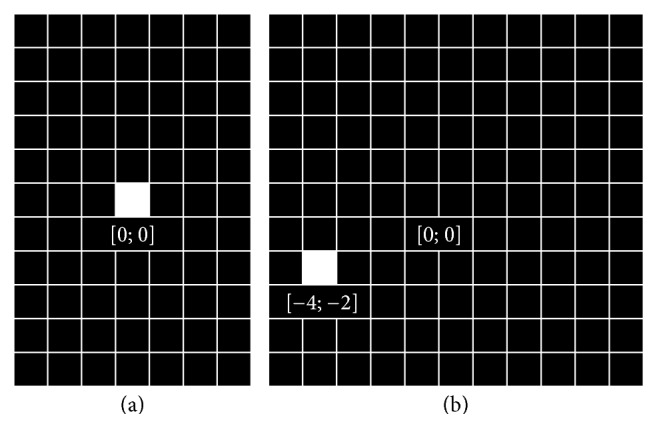
The main idea of phase correlation: on the left: the phase correlation of the function with itself, the *δ*-distribution *δ*(*x*; *y*). On the right: the phase correlation of the functions *f*(*x*; *y*) and *f*(*x* − 4; *y* − 2), *δ*-distribution *δ*(*x* + 4; *y* + 2). It is necessary to shift the function *f*(*x* − 4; *y* − 2) by vector (−4; −2) for further image processing and reconstruction.

In an illustration of the *δ*-distribution, maximum pixel value is used instead of infinity. The illustration of the phase correlation of a function with itself can be seen in Figure 7 on the left.

If two functions are shifted in arguments, that is, *f*_2_(*x*; *y*) = *f*_1_(*x* − *x*_0_; *y* − *y*_0_), their Fourier transforms are shifted in phase; that is,

(10) $$\begin{array}{c} F_{2}\left(\;\xi ;\eta \;\right)=F_{1}\left(\;\xi ;\eta \;\right)\cdot \exp\left(\;-i\left(\;\xi x_{0}+\eta y_{0}\;\right)\;\right) \end{array}$$

and their phase correlation function is the *δ*-distribution shifted in arguments by the opposite shift vector

(11) Pf1;f2x;yF−1exp⁡iξx0+ηy0=δx+x0;y+y0.

The illustration of phase correlation of shifted but otherwise identical images can be seen in Figure 7 on the right.

This is the main idea of phase correlation. The task to find a shift between two images is converted by the phase correlation to the task of finding the only nonzero point in a matrix (computation using the discrete Fourier transform). If the images are not identical (up to a shift), that is, if the images are not ideal, the phase correlation function is more complicated, but it still has a global maximum at the coordinates corresponding to the shift vector. To keep this maximum global, (6) can be modified with possibilities suggested in (7) or modifying directly the original images and the parameters of these modifications can be optimized.

### 2.5. Rotated Images

The phase correlation function can be also used for estimation of image rotation and rescale. The method was first published by Reddy and Chatterji [20]. Let *f*_2_ be function *f*_1_ rotated and shifted in arguments; that is,

(12) f2x;y=f1xcos⁡θ−ysin⁡θ−x0;xsin⁡θ+ycos⁡θ−y0.

Their Fourier spectra and amplitude spectra are related as follows:

(13) F2ξ;η=exp⁡−iξx0+ηy0·F1ξcos⁡θ−ηsin⁡θ;ξsin⁡θ+ηcos⁡θ,A2ξ;η=A1ξcos⁡θ−ηsin⁡θ;ξsin⁡θ+ηcos⁡θ.

The shift results in a phase shift and the spectra are rotated in the same way as the original functions. A crucial step here is transformation of the amplitude spectra into the polar coordinate system to obtain functions *A*_1_^*p*^; *A*_2_^*p*^ : *ℝ*_0_^+^ × 〈0; 2*π*) → *ℝ*_0_^+^ such that *A*_1_^*p*^(*ρ*; *φ*) = *A*_2_^*p*^(*ρ*; *φ* + *θ*). The rotation around an unknown centre of rotation was transformed to a shift. This shift is estimated with the standard phase correlation, Section 2.4.; after rotating back by the measured angle, the shift (*x*_0_; *y*_0_) is measured with another computation of the phase correlation.

### 2.6. Scaled Images

Let *f*_2_ be function *f*_1_ rotated, shifted, and scaled in arguments; that is,

(14) f2x;y=f1αxcos⁡θ−ysin⁡θ−x0;αxsin⁡θ+ycos⁡θ−y0.

Their Fourier spectra and amplitude spectra are related as follows:

(15) F2ξ;η=1α2exp⁡−iξx0+ηy0·F11αξcos⁡θ−ηsin⁡θ;1αξsin⁡θ+ηcos⁡θ,A2ξ;η=1α2·A11αξcos⁡θ−ηsin⁡θ;1αξsin⁡θ+ηcos⁡θ.

The shift results in a phase shift; the spectra are rotated in the same way as the original functions and scaled with a reciprocal factor. A crucial step here is transformation of the amplitude spectra into the logarithmic-polar coordinate system

(16) $$\begin{array}{c} \exp\rho =\sqrt{x^{2}+y^{2}};\;x=\exp\rho \cos\varphi ;\;\;y=\exp\rho \sin\varphi \end{array}$$

to obtain *A*_1_^*p*^; *A*_2_^*p*^ : *ℝ*_0_^+^ × 〈0; 2*π*) → *ℝ*_0_^+^ such that *A*_2_^1*p*^(*ρ*; *φ*) = *A*_2_^1*p*^(*ρ* − ln⁡*α*; *φ* + *θ*).

Both rotation and scale changes were transformed to a shift. The unknown angle *θ* and unknown factor *α* can be estimated by means of the phase correlation applied on the amplitude spectra in the logarithmic-polar coordinate system *A*_1_^1*p*^; *A*_2_^1*p*^. After rotating function *f*_2_ back by the estimated angle *θ* and scaling by factor *α*, the shift vector (*x*_0_; *y*_0_) is estimated by means of the standard phase correlation, Section 2.4.

### 2.7. Practical Issues

Amplitude spectra of real functions are even functions *A*(*ξ*; *η*) = *A*(−*ξ*; −*η*); therefore it is sufficient to use only a half of the domain of the spectra, for example, *ξ* ≥ 0. If amplitude spectra (computed by means of the discrete Fourier transform) are transformed to polar coordinates, only a half of the domain on the angular axis is sufficient.

The amplitude spectra have very high values in [0; 0] and its close neighbourhood compared to the rest of the domain; therefore instead of the values of the amplitude spectra it is better to use their logarithms ln⁡(1 + *A*_1_(*ξ*; *η*)); ln⁡(1 + *A*_2_(*ξ*; *η*)) to use the dynamic range of the amplitude spectra more effectively.

The discrete Fourier transform takes images as if they were periodic with period *N* on both axes. The image edges thus represent a jump in pixel values. Therefore, it is necessary to “remove” image edges, to smooth them out by multiplying them with so-called windowing functions. The most common are Gaussian and Hanning window functions. Most commonly they are applied radial-symmetrically. If there are important structures closer to image corners, they may also keep untouched a square or a rectangle and then decrease to zero.

Image pixel coordinates are integers but the scaling, rotation, and shift vector are obviously stated as not-integer values by registration. Therefore, values of pixels in the target image are calculated by various interpolation methods (nearest neighbour, bilinear or bicubic interpolation).

## 3. Results

The theory described in the previous section was applied to a series of 43 partially focused images of a sandstone sample (locality Brno-Hády, Czech Republic) which was acquired using a central projection in the viewing field 2,5 × 1,875 cm. The results are summarized in Table 1. We have identified mostly insignificant rotation (note that rotation of two hundred arc seconds around the centre means deviation of one-half pixel in the image with the resolution 1024 × 768); shift of the image centre is more significant.

**Figure 8:**
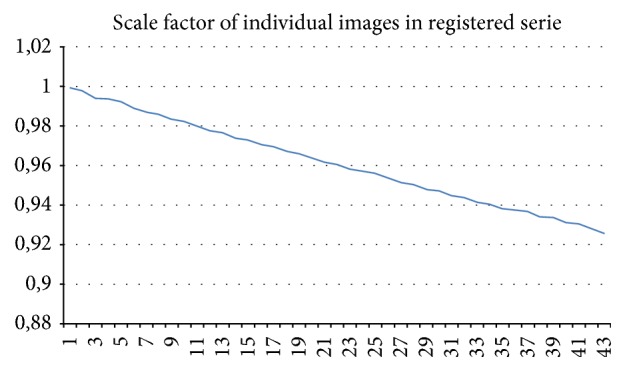
Scaling of individual images in processed serie, dependence is not precisely linear.

**Figure 9:**
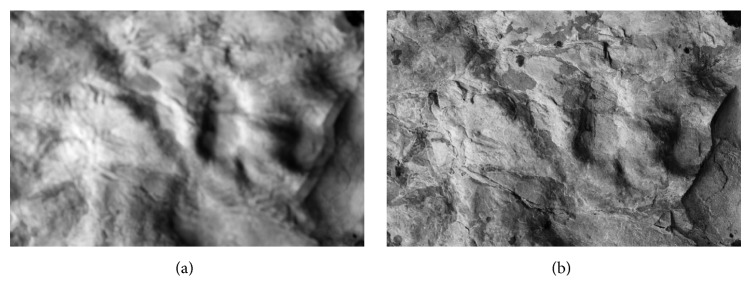
Sum of first, tenth, twentieth, and fortieth partially focused photo of sandstone sample aquired in central projection: (a) no registration, (b) registration described in section 2 (first and forty-third image of this serie we can see in Figure 5).

The graph in Figure 8 illustrates the scaling for the individual image (relative to the first). It is not precisely linear in practice as was stated in Section 2.1 (see also Figure 6). In Figure 9 we can see the sum of four input images without registration (a) and the sum of the same images preprocessed using the registration method described in Section 3 (b).

The entire process of 2D and 3D processing of the series of the partially focused images acquired in central projection thus proceeds as follows:

- Preprocessing: registration of partially focused images using elementary mathematics (see Figure 6 on the left) simple but usually not precise enough method; or registration using the method proposed in Sections 2.2–2.7 in this paper.
- 2D reconstruction: Identification of sharp parts in separated images and composition of a new whole sharp 2D image (see [3, 4]).
- 3D reconstruction: Height is assigned to all the image points (see [1, 2, 4, 10]).

**Figure 10:**
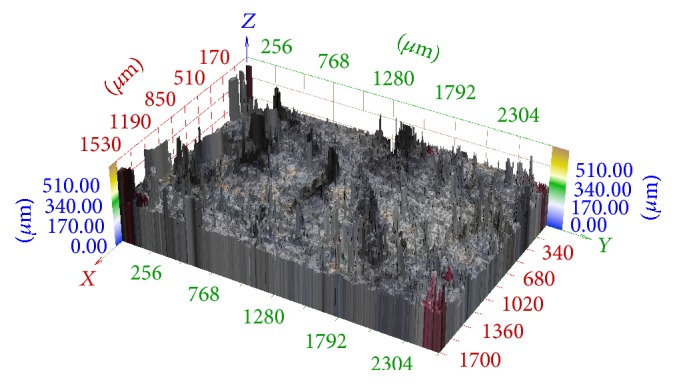
3D reconstruction of image series aquired in central projection without any registration.

In Figure 10 we can see the 3D reconstruction of this series using the method described in [1, 2], that is, without any registration. The registration is not necessary in the case of confocal microscope images. However, images differ in scaling and rotation in the case of classic cameras and their 3D reconstruction without any registration is unusable.

**Figure 11:**
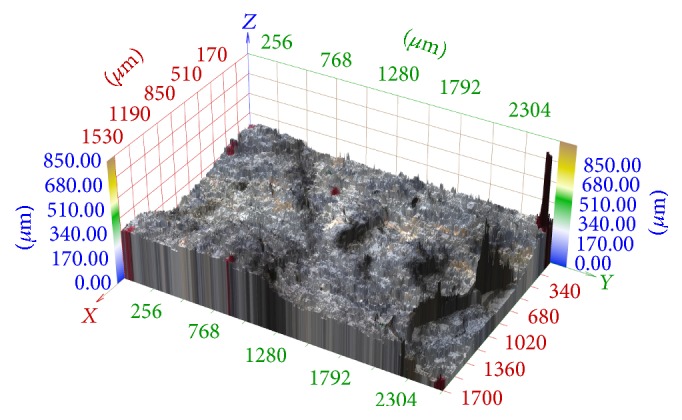
3D reconstruction of image series aquired in central projection registered using elementary methods according to Figure 6 on the left.

In Figure 11, we can see the 3D reconstruction of the same series which was transformed using elementary mathematics according to Figure 6 on the left. The result is significantly better but artifacts are still evident.

**Figure 12:**
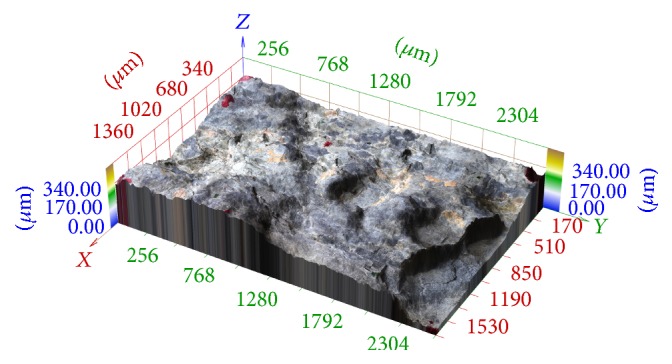
3D reconstruction of image series aquired in central projection registered by phase correlation described in section 2.

In Figure 12, there is illustrated the 3D reconstruction of the same series which was registered using phase correlation described in Section 2. No artifacts are perspicuous in this reconstruction.

## 4. Conclusions

In the case of 3D reconstruction of a series of partially focused images of oversized surfaces, we usually cannot neglect the angle between projection rays and therefore even the different scale of individual images. In the simplest case, we can assume that the scaling is linear and no other geometric transformations occur. This case can be solved using elementary methods but subsequent 3D reconstruction contains usually unwanted artifacts. In real devices, scaling is not linear and images can be shifted and even rotated with respect to each other. If accurate 3D reconstruction is required, precise image registration is necessary as preprocessing. Phase correlation is a suitable method for this preprocessing. It is able to detect the above-mentioned transformations with subpixel precision and we can neatly eliminate them.

## Figures and Tables

**Table 1:** Scales, rotations, and shift vectors detected for the second to forty-third image (relative to the first).

Img. number	Scale	Angle (arc sec)	Shift vector (pixels)
2	0.99914	5.49	[−0.3; 0.5]
3	0.99757	14.77	[−0.1; 0.2]
4	0.99398	4.62	[0.3; 0.3]
5	0.99349	14.65	[0.5; 0.2]
6	0.99198	16.98	[0.6; 0.1]
7	0.98878	−6.44	[−1.6; 0.0]
8	0.98694	11.93	[1.7; 0.0]
9	0.98565	20.53	[0.5; 0.1]
10	0.98324	297.58	[0.4; 0.2]
11	0.98206	297.86	[0.4; 0.1]
12	0.97986	302.90	[0.3; 0.1]
13	0.97749	297.52	[0.3; 0.2]
14	0.97636	5.87	[0.1; 0.2]
15	0.97382	3.52	[0.1; 0.3]
16	0.97277	12.75	[0.1; 0.2]
17	0.97069	6.07	[0.0; 0.3]
18	0.96932	5.20	[−0.1; 0.4]
19	0.96716	4.13	[−0.2; 0.5]
20	0.96584	3.33	[−0.2; 0.6]
21	0.96372	3.10	[0.4; 0.7]
22	0.96156	−3.71	[−0.5; 0.7]
23	0.96036	−4.77	[0.2; 0.5]
24	0.95810	5.74	[0.1; 0.8]
25	0.95690	2.63	[0.0; 0.7]
26	0.95579	−14.45	[0.0; 0.5]
27	0.95358	4.72	[−2.3; 0.9]
28	0.95135	−9.62	[0.0; −0.9]
29	0.95020	−302.73	[−2.6; 0.5]
30	0.94808	3.41	[−1.0; 0.2]
31	0.94697	−13.04	[−1.1; 0.3]
32	0.94472	−6.45	[−1.1; 0.5]
33	0.94369	3.45	[−1.7; 0.5]
34	0.94134	4.95	[−1.6; 0.7]
35	0.94027	−4.22	[−1.1; 1.5]
36	0.93826	−4.97	[−1.3; 1.4]
37	0.93743	3.02	[−1.2; 1.5]
38	0.93669	−8.02	[−1.2; 1.5]
39	0.93419	9.59	[−1.3; 1.5]
40	0.93351	5.97	[−1.7; 1.0]
41	0.93093	11.41	[−1.3; 0.9]
42	0.93023	10.36	[1.3; 0.9]
43	0.92805	14.84	[−1.2; 0.7]
